# Final analyses of OPTiM: a randomized phase III trial of talimogene laherparepvec versus granulocyte-macrophage colony-stimulating factor in unresectable stage III–IV melanoma

**DOI:** 10.1186/s40425-019-0623-z

**Published:** 2019-06-06

**Authors:** Robert H. I. Andtbacka, Frances Collichio, Kevin J. Harrington, Mark R. Middleton, Gerald Downey, Katarina Ӧhrling, Howard L. Kaufman

**Affiliations:** 1 Huntsman Cancer Institute, University of Utah, Salt Lake City, UT USA; 2 University of North Carolina Medical Center, Chapel Hill, NC USA; 3 The Institute of Cancer Research/The Royal Marsden Hospital NIHR Biomedical Research Centre, London, SW3 6JB UK; 4 NIHR Biomedical Research Centre, Oxford, UK; 5 Global Biostatistical Science, Amgen Ltd, Cambridge, UK; 6 Medical development, Amgen (Europe) GmbH, Zug, Switzerland; 7 Massachusetts General Hospital, Boston, MA USA

**Keywords:** Efficacy, Final analysis, Melanoma, Overall survival, Talimogene laherparepvec

## Abstract

**Background:**

Talimogene laherparepvec is an oncolytic immunotherapy approved in the US, Europe, Australia and Switzerland. We report the final planned analysis of OPTiM, a randomized open-label phase III trial in patients with unresectable stage IIIB–IVM1c melanoma.

**Methods:**

Patients were randomized 2:1 to receive intratumoral talimogene laherparepvec or subcutaneous recombinant GM-CSF. In addition to overall survival (OS), durable response rate (DRR), objective response rate (ORR), complete responses (CR), and safety are also reported. All final analyses are considered to be descriptive and treatment responses were assessed by the investigators.

**Results:**

Of 436 patients in the intent-to-treat population, 295 were allocated to talimogene laherparepvec and 141 to GM-CSF. Median follow-up in the final OS analysis was 49 months. Median OS was 23.3 months (95% confidence interval [CI], 19.5–29.6) and 18.9 months (95% CI, 16.0–23.7) in the talimogene laherparepvec and GM-CSF arms, respectively (unstratified hazard ratio, 0.79; 95% CI, 0.62–1.00; *p* = 0.0494 [descriptive]). DRR was 19.0 and 1.4% (unadjusted odds ratio, 16.6; 95% CI, 4.0–69.2; *p* < 0.0001); ORR was 31.5 and 6.4%. Fifty (16.9%) and 1 (0.7%) patient in the talimogene laherparepvec and GM-CSF arms, respectively, achieved CR. In talimogene laherparepvec-treated patients, median time to CR was 8.6 months; median CR duration was not reached. Among patients with a CR, 88.5% were estimated to survive at a 5-year landmark analysis. Talimogene laherparepvec efficacy was more pronounced in stage IIIB–IVM1a melanoma as already described in the primary analysis. The safety reporting was consistent with the primary OPTiM analysis.

**Conclusions:**

In this final planned OPTiM analysis, talimogene laherparepvec continued to result in improved longer-term efficacy versus GM-CSF and remained well tolerated. The final analysis also confirms that talimogene laherparepvec was associated with durable CRs that were associated with prolonged survival.

**Trial registration:**

ClinicalTrials.gov identifier: NCT00769704.

**Electronic supplementary material:**

The online version of this article (10.1186/s40425-019-0623-z) contains supplementary material, which is available to authorized users.

## Background

Talimogene laherparepvec (T-VEC), a first-in-class viral oncolytic immunotherapy [1], was approved in the United States in 2015 for the local treatment of unresectable, cutaneous, subcutaneous and nodal lesions in patients with melanoma recurrent after initial surgery [2], based on data from OPTiM, a randomized phase III open-label trial [3–5]. Intratumoral T-VEC significantly improved durable response rate (DRR) versus subcutaneous GM-CSF; achieving a durable response (DR; response lasting ≥6 months) was associated with clinical benefits such as overall survival (OS) and quality of life [3]. In the primary analysis of OS (secondary endpoint performed after 290 deaths; median follow-up 44 months), T-VEC showed a numerically reduced risk of death versus GM-CSF (median OS 18.9 vs. 23.3 months; hazard ratio [HR] 0.79; 95% confidence interval [CI], 0.62–1.00; *P* = 0.051) [4]. Exploratory analyses of OPTiM revealed that the benefit of T-VEC versus GM-CSF (in terms of DRR, objective response rate [ORR] and OS) was more pronounced in patients with stage IIIB–IVM1a disease than in later-stage metastatic disease [4, 5]. A tolerable safety profile for T-VEC, with a low rate of grade 3/4 adverse events (AEs), was also observed [4]. In Europe, T-VEC was approved in 2015 for unresectable, regionally or distantly metastatic (stage IIIB–IVM1a) melanoma with no bone, brain, lung or other visceral disease [6].

Here we report the final planned analysis of OS in the OPTiM trial performed 3 years after the last patient was randomized. Findings are described for the overall intent-to-treat (ITT) population as well as in patients with early metastatic disease (stage IIIB–IVM1a melanoma) included in the European label. Final analyses of objective response, including exploratory analyses of complete responders, are also discussed.

## Methods

### Patients

Eligibility criteria for the OPTiM trial have been described previously [4]. Eligible patients were ≥ 18 years old and had histologically confirmed, unresectable, bidimensionally measurable stage IIIB/C/IV melanoma according to the 7th edition AJCC staging system [7] with ≥1 cutaneous, subcutaneous or nodal lesions that was suitable for direct or ultrasound-guided injection. Other inclusion criteria included an Eastern Cooperative Oncology Group (ECOG) performance status of ≤1; serum lactate dehydrogenase ≤1.5 × upper limit of normal; ≤3 visceral lesions (excluding lung or nodal lesions associated with visceral organs) with none > 3 cm; and adequate organ function. Further details are provided in the Additional file 1.

### Study design and treatment

OPTiM (ClinicalTrials.gov NCT00769704) enrolled subjects at 64 sites in the United States, the United Kingdom, Canada, and South Africa between 2009 and 2011. Patients were randomized in a 2:1 ratio to receive intratumoral T-VEC (at the approved dose [6]) or subcutaneous recombinant GM-CSF [4] (see Additional file 1). Treatment continued for 6 months regardless of the occurrence of progressive disease (unless alternative therapy was clinically indicated). After 6 months, treatment was continued until clinically relevant disease progression, intolerability, consent withdrawal, complete remission, lack of response by 12 months or (T-VEC arm only) disappearance of injectable lesions. Patients with stable or responding disease at 12 months could continue treatment for 6 additional months. Data cut-off for this final analysis of OPTiM was 5 September 2014.

### Assessments and endpoints

#### Efficacy

Efficacy assessments reported here include analysis of DRR, ORR, disease control rate (DCR) and OS using the final OPTiM data set.

Clinical response was evaluated using the modified World Health Organization criteria [8], as previously described [4]. For the primary OPTiM analysis, patients with an ORR (complete response [CR] or partial response [PR]) per investigator were evaluated by a blinded endpoint-assessment committee [5]. After the primary analysis, only response assessments per investigator were collected (and are reported herein). DRR was defined as the rate of CR or PR lasting continuously for ≥6 months and onset within ≤12 months of randomization. DCR was the proportion of patients with CR, PR or stable disease.

An analysis of complete responders in the T-VEC arm was conducted, including the time to achieve CR, the duration of CR once achieved and factors predictive of CR. Analyses also evaluated associations between CR and OS, CR and recurrence-free survival (RFS; date of CR to the date of recurrence, death due to disease progression, or initiation of new anti-melanoma therapy after achieving CR) and CR and treatment-free interval (TFI, time from the last dose of study therapy to first dose of subsequent therapy or censoring in the absence thereof at end of follow-up).

OS was defined as the time from random assignment to death from any cause. Subgroup analyses were performed to investigate the relative effects of treatment on OS according to key covariates, including age, sex, disease stage, tumor burden, ECOG performance status, and line of therapy.

### Safety

Safety was evaluated from enrollment up to the data cut-off for the final analysis using National Cancer Institute Common Terminology Criteria for AEs (CTCAE) version 3.0.

### Statistical analyses

Sample size was determined as described previously [4]. All efficacy analyses reported here are descriptive, as multiple comparisons were not controlled for. Final analysis of DRR was conducted using a 2-sided unadjusted Fisher exact test. Univariate and multivariate analyses (including a logistic regression model) were conducted to identify independent factors associated with achieving CR. The final descriptive analysis of OS was planned to occur 3 years after the last randomization in OPTiM, and used an unadjusted log-rank test and a Cox proportional hazard model to estimate the unstratified HR for treatment effect. Five-year survival in the T-VEC arm was estimated using the Kaplan-Meier method. Exploratory subgroup analyses of OS by key covariates was carried out using the Gail and Simon quantitative interaction test. Full details of the statistical analyses can be found in the Additional file 1.

## Results

### Patients

Of the 436 patients in the ITT population, 295 (68%) were allocated to receive T-VEC and 141 (32%) to GM-CSF. Baseline characteristics, which have been previously reported [4], were generally well balanced between treatment arms (see Additional file 1). Median (range) duration of treatment was 23.1 weeks (0.1–176.7) in the T-VEC arm and 10.0 weeks (0.6–120.0) in the GM-CSF arm. Median follow-up (time from random assignment to analysis) in the final analysis of OS was 49 months.

### Efficacy in the final OPTiM analysis dataset

#### Intent-to treat population (stage IIIB–IVM1c melanoma)

**Fig. 1:**
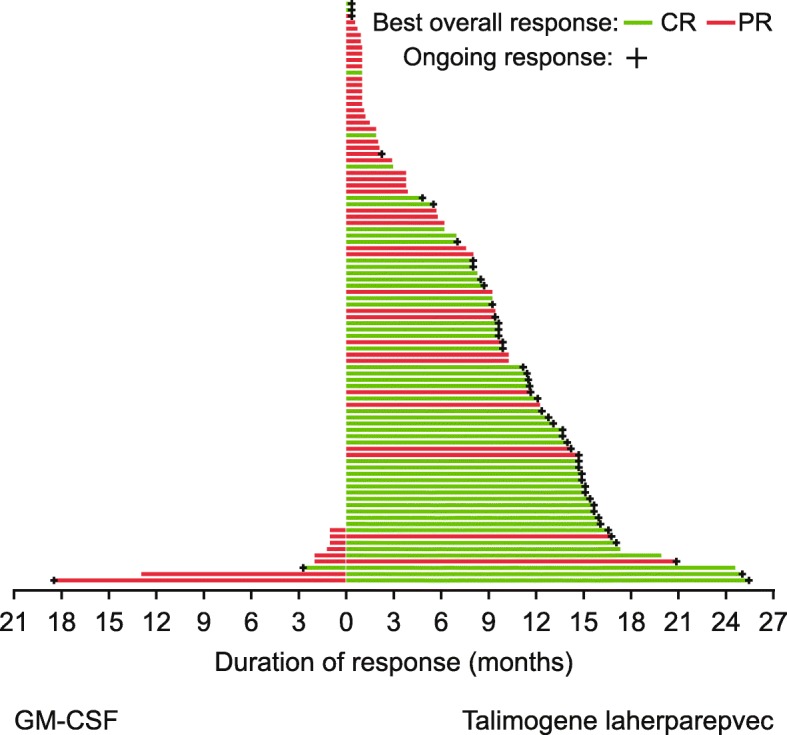
Duration of response for all patients with response per investigator assessment. Duration of response is defined as the longest individual period from entering response (PR or CR) to the first documented evidence of the patient no longer meeting the criteria for being in response or death, whichever is earlier. CR, complete response; GM-CSF, granulocyte-macrophage colony-stimulating factor; PR, partial response

DRR was higher with T-VEC than GM-CSF: 57 (19.3%) and 2 (1.4%) patients, respectively, experienced a durable response per investigator assessment (unadjusted odds ratio, 16.6; 95% CI, 4.0–69.2; *p* < 0.0001). ORR was also higher with T-VEC (31.5%; 95% CI, 26.3–37.2) than GM-CSF (6.4%; 95% CI, 3.0–11.8; Table 1). Overall, 50 (16.9%) and 1 (0.7%) patients in the T-VEC and GM-CSF arms, respectively, achieved CR, while 43 (14.6%) and 8 (5.7%) achieved PR (Table 1). Figure 1 illustrates response duration. The DCR was 76.3% versus 56.7% with T-VEC and GM-CSF, respectively (Table 1).

**Table 1:** Efficacy outcomes in final analysis data set of OPTiM

	Talimogene laherparepvec (*n* = 295)	GM-CSF (*n* = 141)	Descriptive *P*-value^a^	Difference	
				%	95% CI^b^
Response per investigator assessment in the intent-to-treat population (Stage IIIB–IVM1c disease)					
DRR, n (%)	57 (19.3)	2 (1.4)	< 0.0001	17.9	12.0–23.1
CR, n (%)	50 (16.9)	1 (0.7)	–	–	–
PR, n (%)	43 (14.6)	8 (5.7)	–	–	–
ORR, % (95% CI)^b^	31.5 (26.3–37.2)	6.4 (3.0–11.8)	< 0.0001	25.1	17.4–31.7
SD, n (%)	132 (44.7)	71 (50.4)	–	–	–
DCR, n (%)	225 (76.3)	80 (56.7)	–	19.5	9.7–29.3
Progressive disease, n (%)	62 (21.0)	42 (29.8)	–	–	–
Not assessed, n (%)	8 (2.7)	19 (13.5)	–	–	–
Estimated OS probability in the intent-to-treat population (Stage IIIB–IVM1c disease), % (95% CI)					
At 12 months	73.7 (68.3–78.4)	69.1 (60.6–76.2)	–	4.6	−4.7–13.8
At 24 months	49.8 (44.0–55.4)	40.3 (32.0–48.4)	–	9.5	−0.5–19.6
At 36 months	38.9 (33.3–44.4)	30.4 (22.9–38.3)	–	8.4	−1.2–18.0
At 48 months	34.5 (28.9–40.1)	23.9 (16.8–31.7)	–	10.6	1.2–20.0
At 60 months	33.4 (27.7–39.2)	NE	–	NE	NE
DRR, ORR, CR and DCR per investigator assessment according to disease stage					
DRR, n/N (%)					
IIIB/C	29/88 (33.0)	0/43 (0)	< 0.0001	33.0	19.1–43.9
IVM1a	18/75 (24.0)	0/43 (0)	0.0003	24.0	10.5–35.5
IIIB–IVM1a	47/163 (28.8)	0/86 (0)	< 0.0001	28.8	20.3–36.5
IVM1b	4/64 (6.3)	1/26 (3.8)	1.0000	2.4	−15.8–12.8
IVM1c	6/67 (9.0)	1/29 (3.4)	0.6710	5.5	−11.5–16.2
ORR, n/N (%)					
IIIB/C	46/88 (52.3)	2/43 (4.7)	< 0.0001	47.6	31.1–59.0
IVM1a	29/75 (38.7)	2/43 (4.7)	< 0.0001	34.0	17.6–46.6
IIIB–IVM1a	75/163 (46.0)	4/86 (4.7)	< 0.0001	41.4	30.6–49.9
IVM1b	9/64 (14.1)	2/26 (7.7)	0.5002	6.4	−13.8–19.5
IVM1c	9/67 (13.4)	3/29 (10.3)	1.0000	3.1	−16.3–16.5
CR, n/N (%)					
IIIB/C	31/88 (35.2)	0/43 (0)	–	–	–
IVM1a	15/75 (20.0)	1/43 (2.3)	–	–	–
IIIB–IVM1a	46/163 (28.2)	1/86 (1.2)	–	–	–
IVM1b	2/64 (3.1)	0/26 (0)	–	–	–
IVM1c	2/67 (3.0)	0/29 (0)	–	–	–
PR, n/N (%)					
IIIB/C	15/88 (17.0)	2/43 (4.7)	–	–	–
IVM1a	14/75 (18.9)	1/43 (2.3)	–	–	–
IIIB–IVM1a	29/163 (17.8)	3/86 (3.5)	–	–	–
IVM1b	7/64 (10.9)	2/26 (7.7)	–	–	–
IVM1c	7/67 (10.4)	3/29 (10.3)	–	–	–
DCR, n/N (%)					
IIIB/C	75/88 (85.2)	23/43 (53.5)	–	31.7	13.9–48.6
IVM1a	54/75 (72.0)	24/43 (55.8)	–	16.2	−2.7–34.6
IIIB–IVM1a	129/163 (79.1)	47/86 (54.7)	–	24.5	11.5–37.0
IVM1b	50/64 (78.1)	16/26 (61.5)	–	16.6	−4.9–39.3
IVM1c	46/67 (68.7)	17/29 (58.6)	–	10.0	−11.4–32.2

^a^*P*-values calculated using Fisher’s Exact Test

^b^The Clopper-Pearson method was used to calculate exact CIs for binary endpoints. Wilson’s score method with continuity correction was used to calculate an approximate CI for between-group differences in binary rates

*CI* confidence interval, *CR* complete response, *DCR* disease control rate, *DRR* durable response rate, *GM-CSF* granulocyte-macrophage colony-stimulating factor, *NE* not estimable, *ORR* overall response rate, *OS* overall survival, *PR* partial response

**Fig. 2:**
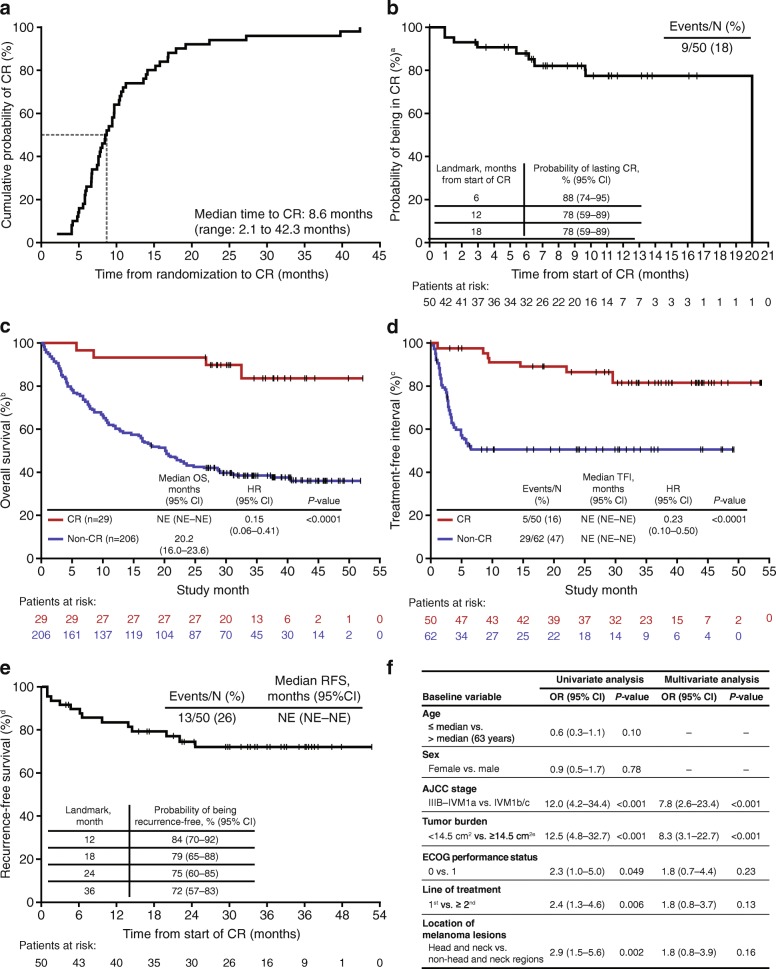
Analyses of CR in stage IIIB–IVM1c melanoma. **a** Time to achieve CR in patients treated with talimogene laherparepvec; **b** Duration of CR in patients treated with talimogene laherparepvec; **c** Kaplan-Meier plot of OS in patients who achieved a CR versus patients who did not achieve a CR prior to a landmark time of 9 months; **d** Kaplan-Meier plot of TFI in patients who achieved a CR versus patients who did not achieve a CR prior to a landmark time of 9 months; **e** RFS after achieving a CR with talimogene laherparepvec; **f** Factors associated with achieving CR with talimogene laherparepvec^f^. ^a^CR duration was defined as the interval from the initial date of CR to the first response of non-CR. Ongoing CRs were censored at the date with a CR. The longest interval was utilized due to multiple CR intervals. Median follow-up for CR duration = 7 months (range < 1 to 20 months). ^b^For landmark analyses, OS was calculated from the landmark time of 9 months after randomization to death. Unadjusted hazard ratios and log-rank *P*-values are shown. ^c^TFI was defined as the interval from the last dose of study therapy and the first dose of systemic therapy categorized as chemotherapy/targeted agent or immunotherapy. The TFI analysis was limited to treated patients with tumor assessments ≥9 months. Unadjusted hazard ratios (HR) and log-rank *P*-values are shown. ^d^RFS after achieving a CR was calculated from date of CR to date of recurrence, death due to disease progression, or start of new anti-melanoma therapy. Median follow-up for RFS = 31 months (range 1 to 53 months). ^e^14.5 cm^2^ was the median tumor burden. ^f^Patients treated with talimogene laherparepvec who achieved CR (*n* = 50) versus those who did not (*n* = 245) using logistic regression models. AJCC, American Joint Committee on Cancer; CI, confidence interval; CR, complete response; ECOG, Eastern Cooperative Oncology Group; HR, hazard ratio; ITT, intent-to-treat; NE, not evaluable; OR, odds ratio; OS, overall survival; RFS, recurrence-free survival; TFI, treatment-free interval

Median time to CR in T-VEC-treated patients was 8.6 months (range, 2.1–42.3; Fig. 2a). Eighteen months after CR was achieved, the probability of remaining in CR was 78% (Fig. 2b). Compared with patients not achieving a CR before a landmark time of 9 months, achieving a CR was associated with an improvement in OS (Fig. 2c) and TFI (Fig. 2d). Among patients with a CR, median OS was not reached, with 88.5% (74–95) estimated to survive at a landmark analysis of 5 years. Overall, 72% of patients who achieved a CR were free from recurrence of melanoma 3 years after achieving a CR (Fig. 2e). Table 2 summarises baseline demographics and disease characteristics in T-VEC-treated patients achieving CR. Following adjustment for potential confounding factors using multivariate analysis, achievement of CR with T-VEC was significantly associated with an earlier stage of metastatic disease (Stage IIIB-IVM1a) and a baseline tumor burden of < 14.5 cm^2^ (Fig. 2f).

**Table 2:** Characteristics of patients treated with talimogene laherparepvec in OPTiM by complete response and partial response (per investigator assessment)^a^

Characteristic	Complete response (*n* = 50)	Partial response (*n* = 43)
Median (IQR) age, years	70 (60–78)	63 (53–77)
Female	20 (40)	21 (49)
ECOG performance status = 0	42 (84)	31 (72)
AJCC stage		
IIIB/C	31 (62)	15 (35)
IVM1a	15 (30)	14 (33)
IIIB–IVM1a	46 (92)	29 (67)
IVM1b	2 (4)	7 (16)
IVM1c	2 (4)	7 (16)
In-transit or distant skin metastases		
IIIB–IVM1a	42 (84)	20 (47)
IIIB–IVM1c	44 (88)	26 (60)
Elevated LDH (>ULN)	0	0
Line of treatment		
1st	33 (66)	27 (63)
≥ 2nd	17 (34)	16 (37)
Median baseline tumor burden (range), cm^2^	4.6 (0.3–38.3)	10.9 (0.6–280.6)
*BRAF* status		
Mutation	5 (10)	9 (21)
Wild type	5 (10)	9 (21)
Unknown/missing	40 (80)	25 (58)
HSV-1 seropositive at baseline	32 (64)	28 (65)

Data presented are number (%) of patients, unless otherwise indicated

^a^Among 295 patients randomized to talimogene laherparepvec, 291 received treatment and 287 were evaluable for response assessment per investigator assessment

*AJCC* American Joint Committee on Cancer, *ECOG* Eastern Cooperative Oncology Group, *HSV* herpes simplex virus, *IQR* interquartile range, *LDH* lactate dehydrogenase, *ULN* upper limit of normal

**Fig. 3:**
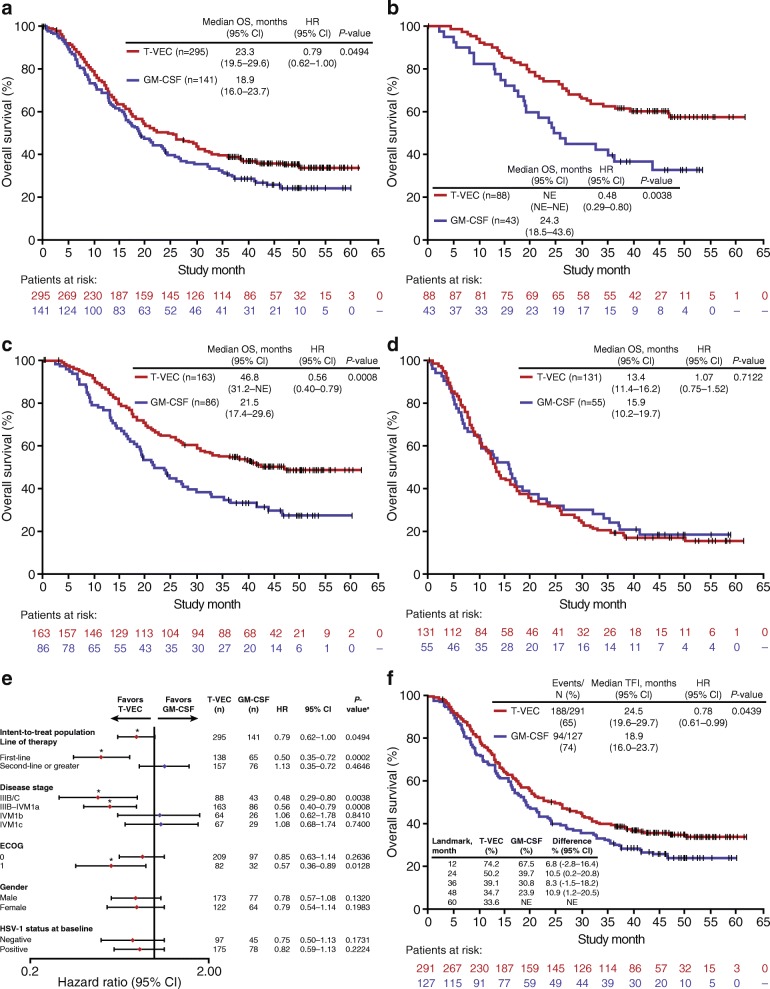
Final planned analyses of OS. **a** Final OS in the intent-to-treat population; **b** Final OS in patients with stage IIIB/C melanoma; **c** Final OS in patients with stage IIIB-IVM1a melanoma; **d** Final OS in patients with stage IVM1b/c melanoma; **e** Exploratory subgroup analyses of final OS (intent-to-treat population); and **f** Final OS in the treatment-received population (all randomized patients excluding those who did not receive allocated treatment; *n* = 4 in the talimogene laherparepvec arm; *n* = 14 in the GM-CSF arm). ^a^*P*-values are descriptive and represent the statistical significance of the treatment difference within the subgroup from log-rank test unless otherwise stated (*P < 0.05). CI, confidence interval; ECOG, Eastern Cooperative Oncology Group; GM-CSF, granulocyte-macrophage colony-stimulating factor; HR, hazard ratio; NE, not estimable; OS, overall survival; T-VEC, talimogene laherparepvec

After five more months of follow-up versus the primary analysis of OS [4], one additional survival event occurred. Median OS was 23.3 months (95% CI, 19.5–29.6) with T-VEC and 18.9 months (95% CI, 16.0–23.7) with GM-CSF (Fig. 3a). Reduction in the risk of death was 21% with T-VEC versus GM-CSF (unstratified HR, 0.79 [95% CI, 0.62–1.00]; *P* = 0.0494 [descriptive]). Estimated 5-year survival in the T-VEC arm was 33.4% (Table 1). Subgroup analyses that were performed to investigate the relative effects of treatment across several key covariates for OS are shown in Fig. 3e. When the 18 patients who did not receive allocated treatment were excluded (T-VEC arm, *n* = 4; GM-CSF arm, *n* = 14), median OS in the final analysis dataset was 24.5 versus 18.9 months for T-VEC versus GM-CSF (HR, 0.78; *P* = 0.0439 [descriptive]; Fig. 3f).

According to an ad-hoc sensitivity analysis for OS accounting for subsequent systemic anti-cancer treatment (including ipilimumab, vemurafenib, dabrafenib, trametinib or an anti-PD-1), there was a 27% reduction in the risk of death for T-VEC versus GM-CSF (unadjusted HR, 0.73; 95% Cl, 0.59–0.92; *P* = 0.0069 [descriptive]).

#### Stage IIIB–IVM1a disease

Effects of T-VEC on DRR, ORR, CR and DCR were more pronounced in stage IIIB–IVM1a melanoma (28.8% DRR, 46.0% ORR and 79.1% DCR) than in more advanced disease (Table 1). Overall, 46/50 (92%) CRs occurred in stage IIIB–IVM1a melanoma (Tables 1 and 2). In T-VEC-treated patients, the CR rate in stage IIIB–IVM1a disease was 28.2% (46/163) and the median time to CR was 8.5 months (range, 2.1–42.3).

Effects of T-VEC on OS were particularly pronounced versus GM-CSF among patients with stage IIIB/C (HR, 0.48, *P* < 0.05 [descriptive]; Fig. 3b) and stage IIIB–IVM1a disease (HR, 0.56; 95% CI, 0.40–0.79; *P* < 0.001 [descriptive]; Fig. 3c) versus the ITT population including in stage IVM1b/c disease (Fig. 3d). Estimated 5-year survival with T-VEC was 48.9% (95% CI, 40.6–56.7) in stage IIIB–IVM1a melanoma versus 15.1% (95% CI, 9.3–22.2) in stage IVM1b/c disease.

### Safety

The most common AEs were fatigue, chills, pyrexia, nausea and influenza-like illness (Additional file 1: Table S1). The incidence of these was highest during the first 3 cycles and subsequently decreased (Additional file 1: Figure S1). Most AEs lasted 2–4 days. Treatment-related grade 3/4 AEs occurred in 33 (11.3%) T-VEC-treated patients and 6 (4.7%) GM-CSF-treated patients. The only grade 3/4 AE occurring in ≥2% of T-VEC-treated patients was cellulitis (2.1%) (Additional file 1: Table S1).

Immune-related AEs were reported in 24/295 T-VEC-treated patients. Vitiligo was the most frequently reported immune-related AE, occurring in 18 (6.2%) T-VEC-treated patients (and one (0.8%) GM-CSF-treated patient). All vitiligo events were grade 1/2. Median time to vitiligo onset in the T-VEC arm was 22 weeks (interquartile range, 9–36). No grade 4 immune-related AEs were reported. Four grade 3 immune-related AEs were observed: glomerulonephritis/renal failure (patient with history of partial nephrectomy due to renal cell carcinoma and streptococcal injection site cellulitis), lupus vasculitis, pneumonitis (patient with a history of ulcerative colitis) and psoriasis (patient with psoriasis history).

The Additional file 1 provides further safety information.

## Discussion

OPTiM was the first phase 3 trial to demonstrate a clinical benefit with an oncolytic immunotherapy in any cancer and the largest randomized controlled trial investigating a therapeutic in unresectable stage IIIB/C melanoma. Following the previously reported analysis of the primary endpoint of DRR [4], patients continued follow-up so that a planned final analysis of OS could be performed 3 years after randomization, as reported here. With 49 months of follow-up, median OS for T-VEC and GM-CSF was in line with the primary analysis [4], and was 4.4 months longer in patients receiving T-VEC than GM-CSF (reduction in the relative risk of death versus GM-CSF: 21%; *P* = 0.0494) [4]. Overall, estimated 5-year survival for the T-VEC arm was 33.4%. In accordance with the primary OPTiM analysis [4], the effects of T-VEC on OS were more pronounced in patients with early metastatic melanoma (stage IIIB–IVM1a), with an estimated 5-year survival of 48.9%.

The final OPTiM data set was further analysed in the 17% of patients treated with T-VEC who achieved a CR. Once achieved, CRs were durable; the median duration was not reached over a median follow-up period of more than 4 years. Additionally, median OS in these patients was not reached and approximately 90% were estimated to be alive at 5 years. Similar to T-VEC, prolonged CR duration (once achieved) and association with survival has been reported in checkpoint inhibitor (CPI) trials [9, 10]. Our analyses showed that early metastatic melanoma (stage IIIB–IVM1a) and lower tumor burden (< 14.5 cm^2^) were independent predictors of achieving a CR. These findings indicate that earlier commencement of an effective treatment is important for resolution of melanoma and, in turn, long-term survival. Recent analyses also demonstrated that less advanced disease and lower tumor burden are associated with achieving a CR with CPIs [9, 10]. The median time to achieve CR among T-VEC-treated patients was 8.6 months. Prior analyses showed that almost half of patients responding to T-VEC monotherapy exhibit progression prior to the response [5, 11]. Although progression prior to response prolongs the time it takes to achieve a response, it does not impact the duration of a response once achieved [11]. Hence, if there are injectable lesion(s) remaining, T-VEC should be continued for ≥6 months unless the patient is not benefitting from treatment or another treatment is required.

As previously demonstrated [4] and confirmed here, T-VEC exhibits a tolerable safety profile with a low rate of grade 3/4 AEs. The incidence of treatment-related grade 3/4 AEs with T-VEC in OPTiM was similar to that reported for anti-programmed cell death protein-1/ligand 1 monotherapy [12–16] and lower than that observed with anti-cytotoxic T-lymphocyte-associated protein-4 monotherapy [14, 17]. However, T-VEC is not associated with the same pattern of serious immune-related AEs reported with CPI therapy, such as thyroid dysfunction, hypophysitis, adrenal insufficiency and autoimmune hepatitis [16, 18–20]. It can take months to see an improvement in these AEs and some may never resolve [16, 18–20].

Interest in the use of T-VEC combined with systemic immunotherapy has arisen due its favorable safety profile, proven efficacy as monotherapy, and potentially complementary mode of action, with an ability to activate a CD8 + -dependent systemic immune response [21]. To date, T-VEC combined with CPIs in melanoma has shown improved efficacy versus CPIs alone without notable additional safety concerns [22–25]. A phase III trial of T-VEC/placebo plus pembrolizumab is underway in unresectable stage IIIB–IVM1c melanoma (MASTERKEY 265; NCT02263508).

Even if the final OS analysis was planned, a limitation is that all final subgroup analyses were descriptive, and some were post-hoc and exploratory. Additionally, after the primary OPTiM analysis, only response assessments per investigator were collected. In contrast, in the primary analysis of OPTiM, the investigator-reported ORRs (CR + PR) were independently evaluated by a blinded endpoint-assessment committee. Nevertheless, the final analyses presented here provide important new practical insights into the use of T-VEC in patients with unresectable stage III–IV melanoma.

In conclusion, as well as demonstrating a longer-term effect on survival, this analysis confirms that T-VEC resulted in high CR rates, most notably in patients with early metastatic melanoma (stage IIIB–IVM1a). Once achieved, CRs were durable and associated with prolonged survival. The favorable clinical outcomes observed in some patients treated with T-VEC, along with its good safety profile, support continued efforts to further define its future role in melanoma as a combination partner with immunotherapy.

## Additional file

Additional file 1: Supplement to: Final analyses of OPTiM: a randomized phase III trial of talimogene laherparepvec versus granulocyte-macrophage colony-stimulating factor in unresectable stage III–IV melanoma (DOC 154 kb)

## Abbreviations

AE: Adverse event
AJCC: American Joint Committee on Cancer
CI: Confidence interval
CR: Complete response
CTCAE: Common Terminology Criteria for Adverse Events
DCR: Disease control rate
DR: Durable response
DRR: Durable response rate
ECOG: Eastern Cooperative Oncology Group
GM-CSF: Granulocyte-macrophage colony-stimulating factor
HR: Hazard ratio
ITT: Intent-to-treat
OR: Odds ratio
ORR: Objective response rate
OS: Overall survival
PR: Partial response
RFS: Recurrence-free survival
TFI: Treatment-free interval
T-VEC: Talimogene laherparepvec

## References

[1] Kohlhapp FJ, Kaufman HL (2016). Molecular pathways: mechanism of action for talimogene laherparepvec, a new oncolytic virus immunotherapy. Clin Cancer Res.

[2] U. S. Food and Drug Administration (2015). Imlygic (talimogene laherparepvec) prescribing information.

[3] Kaufman HL, Andtbacka RHI, Collichio FA, Wolf M, Zhao Z, Shilkrut M (2017). Durable response rate as an endpoint in cancer immunotherapy: insights from oncolytic virus clinical trials. J Immunother Cancer.

[4] Andtbacka RH, Kaufman HL, Collichio F, Amatruda T, Senzer N, Chesney J (2015). Talimogene laherparepvec improves durable response rate in patients with advanced melanoma. J Clin Oncol.

[5] Harrington KJ, Andtbacka RH, Collichio F, Downey G, Chen L, Szabo Z (2016). Efficacy and safety of talimogene laherparepvec versus granulocyte-macrophage colony-stimulating factor in patients with stage IIIB/C and IVM1a melanoma: subanalysis of the phase III OPTiM trial. Onco Targets Ther.

[6] European Medicines Agency (2015). Summary of product characteristics for Imlygic.

[7] American Joint Committee on Cancer. AJCC cancer staging handbook: from the AJCC cancer staging manual. 7th ed. New York: Springer; 2010.

[8] World Health Organization (1979). WHO handbook for reporting results of cancer treatment.

[9] Robert C, Larkin J, Ascierto PA, Long GV, Hassel JC, Schadendorf D, et al. 1213O Characterization of complete responses (CRs) in patients with advanced melanoma (MEL) who received the combination of nivolumab (NIVO) and ipilimumab, or nivolumamb alone. Ann Oncol. 2017;28(Suppl 5):mdx377.

[10] Robert C, Ribas A, Hamid O, Daud A, Wolchok JD, Joshua AM (2018). Durable complete response after discontinuation of pembrolizumab in patients with metastatic melanoma. J Clin Oncol.

[11] Andtbacka RH, Ross M, Puzanov I, Milhem M, Collichio F, Delman KA (2016). Patterns of clinical response with talimogene laherparepvec (T-VEC) in patients with melanoma treated in the OPTiM phase III clinical trial. Ann Surg Oncol.

[12] Hamid O, Robert C, Daud A, Hodi FS, Hwu W-J, Kefford R (2013). Safety and tumor responses with lambrolizumab (anti–PD-1) in melanoma. N Engl J Med.

[13] Ribas A, Puzanov I, Dummer R, Schadendorf D, Hamid O, Robert C (2015). Pembrolizumab versus investigator-choice chemotherapy for ipilimumab-refractory melanoma (KEYNOTE-002): a randomised, controlled, phase 2 trial. Lancet Oncol.

[14] Robert C, Schachter J, Long GV, Arance A, Grob JJ, Mortier L (2015). Pembrolizumab versus ipilimumab in advanced melanoma. N Engl J Med.

[15] Weber JS, D'Angelo SP, Minor D, Hodi FS, Gutzmer R, Neyns B (2015). Nivolumab versus chemotherapy in patients with advanced melanoma who progressed after anti-CTLA-4 treatment (CheckMate 037): a randomised, controlled, open-label, phase 3 trial. Lancet Oncol.

[16] Naidoo J, Page DB, Li BT, Connell LC, Schindler K, Lacouture ME (2015). Toxicities of the anti-PD-1 and anti-PD-L1 immune checkpoint antibodies. Ann Oncol.

[17] Hodi FS, O'Day SJ, McDermott DF, Weber RW, Sosman JA, Haanen JB (2010). Improved survival with ipilimumab in patients with metastatic melanoma. N Engl J Med.

[18] Weber JS, Dummer R, de Pril V, Lebbe C, Hodi FS (2013). Patterns of onset and resolution of immune-related adverse events of special interest with ipilimumab: detailed safety analysis from a phase 3 trial in patients with advanced melanoma. Cancer.

[19] McGettigan S, Rubin KM. PD-1 inhibitor therapy: consensus statement from the faculty of the Melanoma Nursing Initiative on managing adverse events. Clin J Oncol Nurs. 2017;21(Suppl 4):42–51. 10.1188/17.CJON.S4.42-51 28738055

[20] Gonzalez-Rodriguez E, Rodriguez-Abreu D (2016). Immune checkpoint inhibitors: review and management of endocrine adverse events. Oncologist.

[21] Moesta AK, Cooke K, Piasecki J, Mitchell P, Rottman JB, Fitzgerald K (2017). Local delivery of OncoVEX(mGM-CSF) generates systemic antitumor immune responses enhanced by cytotoxic t-lymphocyte-associated protein blockade. Clin Cancer Res.

[22] Long GV, Dummer R, Ribas A, Puzanov I, VanderWalde A, Andtbacka RH (2016). Efficacy analysis of MASTERKEY-265 phase 1b study of talimogene laherparepvec (T-VEC) and pembrolizumab (pembro) for unresectable stage IIIB-IV melanoma. J Clin Oncol.

[23] Chesney Jason, Puzanov Igor, Collichio Frances, Singh Parminder, Milhem Mohammed M., Glaspy John, Hamid Omid, Ross Merrick, Friedlander Philip, Garbe Claus, Logan Theodore F., Hauschild Axel, Lebbé Celeste, Chen Lisa, Kim Jenny J., Gansert Jennifer, Andtbacka Robert H.I., Kaufman Howard L. (2018). Randomized, Open-Label Phase II Study Evaluating the Efficacy and Safety of Talimogene Laherparepvec in Combination With Ipilimumab Versus Ipilimumab Alone in Patients With Advanced, Unresectable Melanoma. Journal of Clinical Oncology.

[24] Puzanov I, Milhem MM, Minor D, Hamid O, Li A, Chen L (2016). Talimogene laherparepvec in combination with ipilimumab in previously untreated, unresectable stage IIIB-IV melanoma. J Clin Oncol.

[25] Ribas A, Dummer R, Puzanov I, VanderWalde A, Andtbacka RHI, Michielin O (2017). Oncolytic virotherapy promotes intratumoral T cell infiltration and improves anti-PD-1 immunotherapy. Cell.

