# Supplementary material for: Final analyses of OPTiM: a randomized phase III trial of talimogene laherparepvec versus granulocyte-macrophage colony-stimulating factor in unresectable stage III–IV melanoma
Source: J Immunother Cancer. 2019 Jun 6;7:145. doi: 10.1186/s40425-019-0623-z (PMC6554874; doi:10.1186/s40425-019-0623-z)
Supplement: Supplementary file 1 — Supplement to: Final analyses of OPTiM: a randomized phase III trial of talimogene laherparepvec versus granulocyte-macrophage colony-stimulating factor in unresectable stage III–IV melanoma (DOC 154 kb) [file 40425_2019_623_MOESM1_ESM.doc]

**Supplementary information**

Supplement to: **Final analyses of OPTiM: a randomized phase III trial of talimogene laherparepvec versus granulocyte-macrophage colony-stimulating factor in unresectable stage III–IV melanoma**

Robert H.I. Andtbacka, Frances Collichio, Kevin J. Harrington, Mark R Middleton, Gerald Downey, Katarina Ӧhrling, Howard L. Kaufman

Contents

[Methods 3](#__RefHeading___Toc8212289)

[Key inclusion criteria 3](#__RefHeading___Toc8212290)

[Key exclusion criteria 4](#__RefHeading___Toc8212291)

[Study design and treatment 5](#__RefHeading___Toc8212292)

[Statistical analyses 6](#__RefHeading___Toc8212293)

[Results 7](#__RefHeading___Toc8212294)

[Baseline demographics 7](#__RefHeading___Toc8212295)

[Table S1 8](#__RefHeading___Toc8212296)

[Figure S1. 9](#__RefHeading___Toc8212297)

[Additional safety information 10](#__RefHeading___Toc8212298)

[References 11](#__RefHeading___Toc8212299)

# Methods

## Key inclusion criteria

1. Age ≥ 18 years.
2. Histologically confirmed, not surgically resectable, stage IIIB to IV melanoma suitable for direct or ultrasound-guided injection (at least one cutaneous, subcutaneous, or nodal lesion or aggregation of lesions ≥ 10 mm in diameter).
3. Bidimensionally measurable disease.
4. Serum lactate dehydrogenase ≤1.5 X upper limit of normal.
5. Eastern Cooperative Oncology Group (ECOG) performance status ≤ 1.
6. Adequate organ function.

## Key exclusion criteria

1. Patients requiring intermittent or chronic treatment with an antiviral agent (eg, acyclovir) or high-dose steroids.
2. Primary ocular or mucosal melanoma.
3. Bone metastases.
4. Active cerebral metastases.
5. >3 visceral metastases (except lung or nodal metastases associated with visceral organs), or any visceral metastasis >3 cm; liver metastases had to be stable for ≥ 1 month before random assignment.
6. Use of high-dose steroids.

## Study design and treatment

The first dose of talimogene laherparepvec was given at a dose of 10^6^ pfu/mL to seroconvert herpes simplex virus (HSV)-1-seronegative patients. The second dose of 10^8^ pfu/mL was given 3 weeks later and repeated every 2 weeks thereafter. A maximum total volume of 4.0 mL could be injected at each treatment visit, with per lesion volumes ranging from 0.1 mL for lesions ≤0.5 cm to 4.0 mL for lesions >5 cm in diameter. Granulocyte-macrophage colony-stimulating factor (GM-CSF) was given once daily at a dose of 125 μg/m^2^ for 14 days in 28-day cycles.

Study procedures were approved by the institutional review boards or ethics committees for each participating site, and all patients provided written informed consent.

## Statistical analyses

Final analysis of durable response rate was conducted using a 2-sided unadjusted Fisher exact test. The associations between achieving a complete response (CR) and overall surivial (OS), and between CR and treatment-free interval (estimated using the Kaplan-Meier method), were evaluated using a Cox proportional hazards model and log-rank test in a landmark analysis at 9 months after randomization. The final descriptive analysis of OS was planned to occur 3 years after the last randomization in OPTiM, and used an unadjusted log-rank test and a Cox proportional hazard model to estimate the unstratified hazard ratio (HR) for treatment effect. Five-year survival in the talimogene laherparepvec arm was estimated using the Kaplan-Meier method. Exploratory subgroup analyses of OS by key covariates was carried out using the Gail and Simon quantitative interaction test [1]. Analysis of OS was repeated in the treated population (defined as all randomized patients excluding those that did not receive allocated treatment). The safety population included all patients who received at least 1 (full or partial) dose of study drug. Analyses were performed using SAS software (SAS Institute, Cary, NC).

# Results

## Baseline demographics

Median (range) age was 63 (22–94) and 64 (26–91) years, respectively. Most patients were male (59% and 55% in talimogene laherparepvec and GM-CSF arms, respectively), had an ECOG performance status of 0 (71% and 69%), were HSV-1 seropositive at baseline (59% and 55%), and had received prior therapy (53% and 54%). Previous therapy mostly consisted of chemotherapy, isolated limb perfusion or infusion, radiotherapy, interferon alpha or interleukin 2. Fewer than 5% of patients had previously received ipilimumab, only one patient had been treated with a *BRAF* inhibitor (vemurafenib) and no patients had previously received an anti-programmed cell death protein (PD)-1 inhibitor. A total of 163 patients (55%) in the talimogene laherparepvec arm and 86 patients (61%) in the GM-CSF arm had stage IIIB–IVM1a disease; the remaining patients had more advanced melanoma (stage IVM1b/c).

Table S1. Summary of adverse events in the final OPTiM analysis^a^ (safety analysis set)

|  | **Talimogene laherparepvec (n=292)** | | **GM-CSF (n=127)** | |
| --- | --- | --- | --- | --- |
|  | **Any grade** | **Grade 3–4** | **Any grade** | **Grade 3–4** |
| Fatigue | 148 (50.7) | 5 (1.7) | 46 (36.2) | 1 (0.8) |
| Chills | 144 (49.3) | 0 | 11 (8.7) | 0 |
| Pyrexia | 126 (43.2) | 0 | 11 (8.7) | 0 |
| Nausea | 106 (36.3) | 0 | 25 (19.7) | 0 |
| Influenza-like illness | 90 (30.8) | 2 (0.7) | 19 (15.0) | 0 |
| Injection site pain | 83 (28.4) | 3 (1.0) | 8 (6.3) | 0 |
| Vomiting | 64 (21.9) | 5 (1.7) | 12 (9.4) | 0 |
| Cellulitis | 17 (5.8) | 6 (2.1) | 2 (1.6) | 1 (0.8) |
| Dehydration | 12 (4.1) | 5 (1.7) | 0 | 0 |
| Deep vein thrombosis | 6 (2.1) | 5 (1.7) | 0 | 0 |
| Tumor pain | 23 (7.9) | 5 (1.7) | 7 (5.5) | 0 |

Data presented are number (%) of patients.

^a^Treatment-emergent adverse events of any grade occurring in ≥20% of patients in either arm and/or grade 3 to 4 adverse events occurring in ≥5 patients in either arm

GM-CSF, granulocyte-macrophage colony-stimulating factor.

Figure S1. Incidence of the five most common treatment-emergent adverse events in patients treated with talimogene laherparepvec by treatment cycle^a^


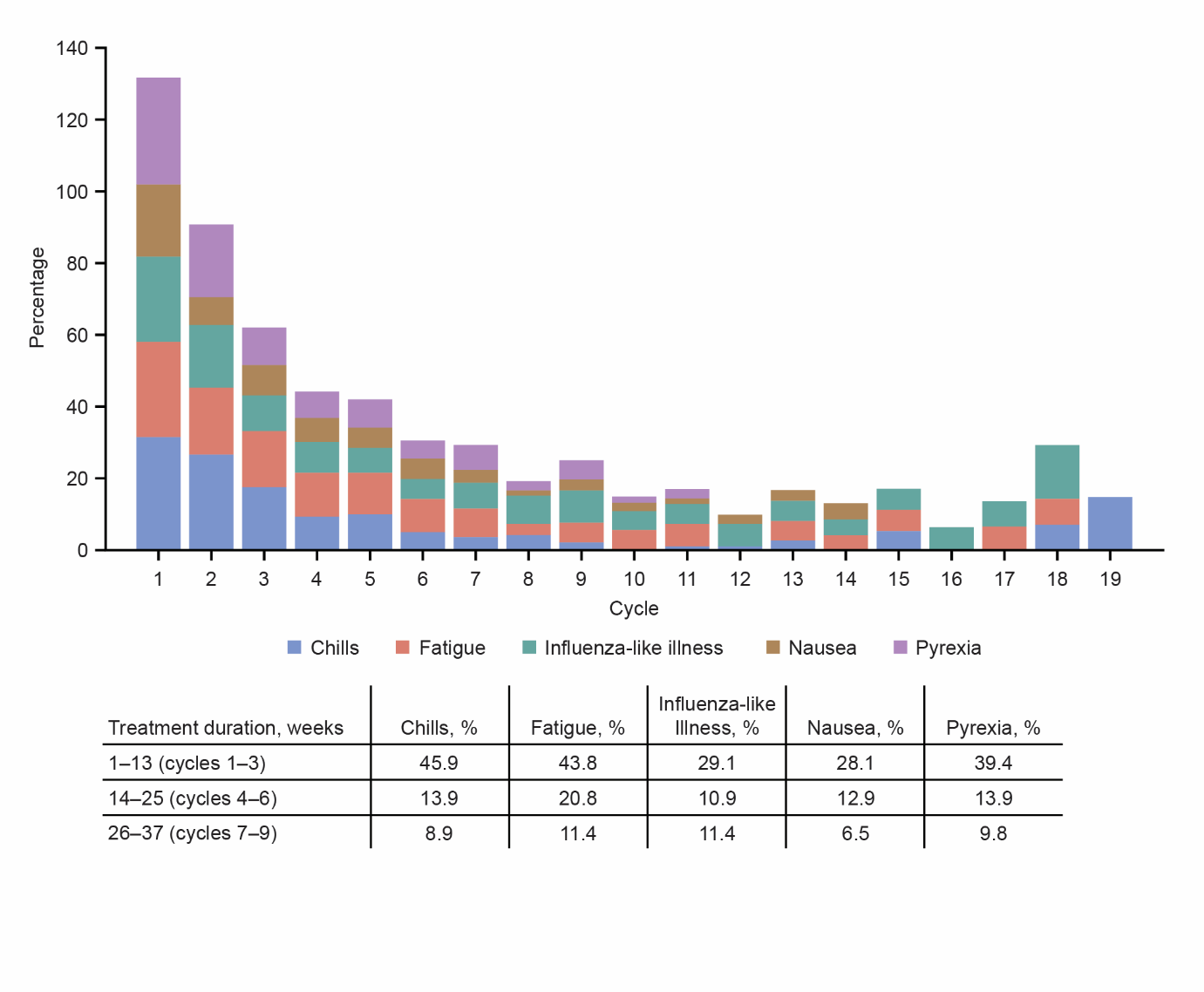


^a^A treatment cycle is defined as two consecutive administrations of talimogene laherparepvec (5 weeks for the first cycle; 4 weeks for each subsequent cycle)

## Additional safety information

Serious adverse events (AEs) of any cause occurred in 80 (27.4%) and 17 (13.4%) patients in the talimogene laherparepvec and GM-CSF arms, respectively. Aside from disease progression (3.4% and 1.6%), the most frequently reported serious AEs were cellulitis (7 [2.4%] and 1 [0.8%]) and pyrexia (5 [1.7%] and 0). AEs leading to discontinuation of study drug occurred in 31 patients (10.6%) and eight patients (6.3%) in the talimogene laherparepvec and GM-CSF arms, respectively.

Of the 12 fatal events in the talimogene laherparepvec arm, none was considered treatment related, and most (8/12) were due to disease progression, except for sepsis (Salmonella infection), myocardial infarction, cardiac arrest (in a patient with pre-existing history of coronary artery disease and arrhythmia), and hypovolemic shock due to bleeding from liver haemangioma. Two fatal non-treatment-related AEs occurred in the GM-CSF arm.

# References

1. Gail M and Simon R. Testing for qualitative interactions between treatment effects and patient subsets. Biometrics 1985;41:361–72.
